# Artificial Intelligence for Detecting COVID-19 With the Aid of Human Cough, Breathing and Speech Signals: Scoping Review

**DOI:** 10.1109/OJEMB.2022.3143688

**Published:** 2022-02-14

**Authors:** Mouzzam Husain, Andrew Simpkin, Claire Gibbons, Tanya Talkar, Daniel Low, Paolo Bonato, Satrajit S. Ghosh, Thomas Quatieri, Derek T. O'Keeffe

**Affiliations:** Health Innovation Via Engineering (HIVE) Lab, Curam, Lero, School of MedicineLambe Institute for Translational ResearchNational University of Ireland Galway 8799 H91 TK33 Galway Ireland; School of Mathematics, Statistics and Applied MathematicsNational University of Ireland 8799 H91 TK33 Galway Ireland; MIT Lincoln Laboratory 57663 Lexington MA 02421 USA; Program in Speech and Hearing Bioscience and TechnologyHarvard Medical School 1811 Boston MA 02115 USA; Program in Speech and Hearing Bioscience and TechnologyHarvard Medical School 1811 Boston MA 02115 USA; MIT McGovern Institute for Brain Research, Cambridge 167631 MA 02139 USA; Department of Physical Medicine and RehabilitationHarvard Medical School, Spaulding Rehabilitation Hospital 1811 Boston MA USA; Health Innovation Via Engineering (HIVE) Lab, Curam, Lero, School of MedicineLambe Institute for Translational ResearchNational University of Ireland Galway 8799 H91 TK33 Galway Ireland; University Hospital Galway, Saolta, Health Services Executive 58040 Ireland

**Keywords:** COVID-19, artificial intelligence, machine learning, cough, speech signals, acoustics, breathing

## Abstract

*Goal:* Official tests for COVID-19 are time consuming, costly, can produce high false negatives, use up vital chemicals and may violate social distancing laws. Therefore, a fast and reliable additional solution using recordings of cough, breathing and speech data for preliminary screening may help alleviate these issues. *Objective:* This scoping review explores how Artificial Intelligence (AI) technology aims to detect COVID-19 disease by using cough, breathing and speech recordings, as reported in the literature. Here, we describe and summarize attributes of the identified AI techniques and datasets used for their implementation. *Methods:* A scoping review was conducted following the guidelines of PRISMA-ScR (Preferred Reporting Items for Systematic Reviews and Meta-Analyses Extension for Scoping Reviews). Electronic databases (Google Scholar, Science Direct, and IEEE Xplore) were searched between 1st April 2020 and 15th August 2021. Terms were selected based on the target intervention (i.e., AI), the target disease (i.e., COVID-19) and acoustic correlates of the disease (i.e., speech, breathing and cough). A narrative approach was used to summarize the extracted data. *Results:* 24 studies and 8 Apps out of the 86 retrieved studies met the inclusion criteria. Half of the publications and Apps were from the USA. The most prominent AI architecture used was a convolutional neural network, followed by a recurrent neural network. AI models were mainly trained, tested and run-on websites and personal computers, rather than on phone apps. More than half of the included studies reported area-under-the-curve performance of greater than 0.90 on symptomatic and negative datasets while one study achieved 100% sensitivity in predicting asymptomatic COVID-19 from cough-, breathing- or speech-based acoustic features. *Conclusions:* The included studies show that AI has the potential to help detect COVID-19 using cough, breathing and speech samples. The proposed methods (with some time and appropriate clinical testing) could prove to be an effective method in detecting various diseases related to respiratory and neurophysiological changes in the human body.

## I. Introduction

**Fig. 1.**
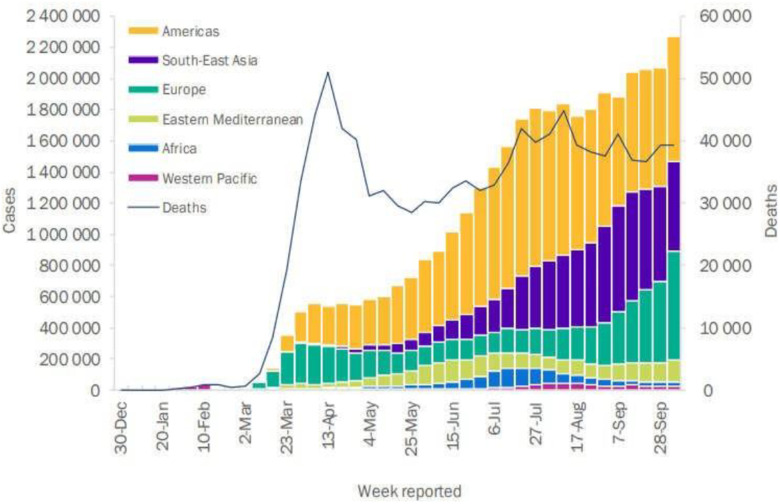
Weekly reports of cases and deaths in various regions [2].

On March 11^th^ 2020, the World Health Organization (WHO) announced that the COVID-19 outbreak had become a pandemic [1]. Fig. 1 depicts the weekly global report by the WHO on the pandemic in various regions from December 2019 to October 2020 [2].

The most common symptoms of COVID-19 include high fever, new cough (may be dry), shortness of breath and loss or change to your sense of smell or taste [3], [4] as well as other neurological effects [5], [6]. Currently, COVID-19 can be detected in two ways: i) Virus detection through nose or throat swabs using a – RT-PCR test and ii) tests which deduce the immune response of the body such as - Antigen and Serology tests [7]. However, multiple limitations are involved with both of these tests. Firstly, high-false negative rates were seen with mass RT-PCR testing [8]. Secondly, tests often require physical contact which may disturb social distancing guidelines. Finally, time and expertise are required for every individual result [9]. Therefore, there is a need to augment the existing techniques to include preliminary screening to reduce these issues.

**Fig. 2.**
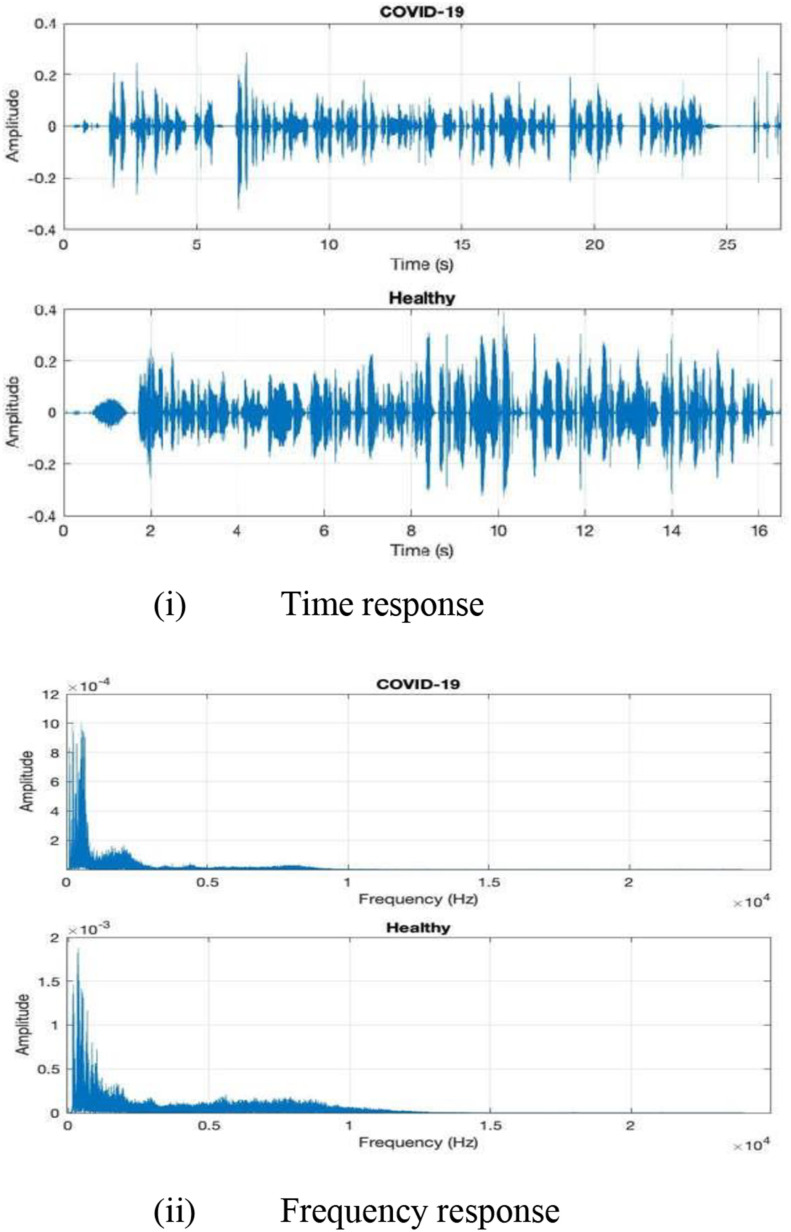
**(i), (ii)**: This figure data was collected and analyzed with ethical approval from University Hospital Galway, Ireland.

Since April 2020, many research organizations began to develop interest in changes to speech and acoustic alterations associated with the virus. Cough being the most common symptom of many diseases, can be differentiated among cases and controls [10] by Artificial Intelligence (AI) and machine learning algorithms. As AI can use deep learning or other machine learning, it could provide better efficiency in detecting viruses in comparison to the recommended tests if it finds novel and predictive patterns [10]. Changes in speech, both read and naturalistic, as due to breathing difficulty, stuffiness and inflammation, likewise showing promise in providing a basis to distinguish healthy from unhealthy cases [11]. The example of speech in Fig. 2 reveals the motivation for using AI as a preliminary screening technique, including better tracking and faster detection of COVID-19. Fig. 2 shows a comparison of a speech waveform and FFT (Fast Fourier transform) of COVID-19 versus healthy cases. The unhealthy subject recording, made soon after a positive COVID-19 diagnosis, exhibits a slower speaking rate (almost half that of the healthy case), greater pause length and duration, less natural and crisp articulation, lower frequency dynamics and high-frequency distribution.

The objective of this review is to examine studies of COVID-19 detection via features derived from speech, breathing and cough recording as presented in the literature. The results may be useful for various institutions developing AI algorithms that use speech signal processing for future pandemics or respiratory-based illnesses.

## II. Methods

To fulfill the aims of this study while certifying an iterative and transparent methodology, a scoping review was conducted following the guidelines of PRISMA-ScR (Preferred Reporting Items for Systematic Reviews and Meta-Analyses Extension for Scoping Reviews) [12]. Below are detailed methods used for this review.

### A. Study Search Resources

In this review, the period chosen for relevant queries was between 1st April 2020 and 15th August 2021. The searches were completed via online databases such as PUBMED, Google Scholar, Science Direct, arXiv and IEEE xplore. In addition, reference lists of included studies were also screened.

### B. Study Search Terminologies

The search terms used were chosen in accordance with the algorithm used, target diseases and symptoms of that disease. The terms were “AI cough”, “machine learning COVID-19”, “coronavirus-19 speech”, “coronavirus-19 cough”, “artificial intelligence for viruses”, “artificial intelligence for coughing” and “COVID-19 breathing”.

### C. Study Eligibility Criteria

The main focus of this review was AI based technology that identifies or predicts the presence of COVID-19 in symptomatic, asymptomatic or negative patients by analyzing recordings of cough, breathing and speech signals. Therefore, studies on non-COVID cough and artificial intelligence alone were excluded. Furthermore, studies involving research or potential use of AI in speech signal processing were also excluded. The selected studies for this review were published in English between 1^st^ April 2020 and 15^st^ August 2021. These include publications, preprints, dissertations, peer-reviewed articles and mobile Apps, excluding overviews, proposals and editorials. This review does not impose any restriction on study design, outcome of the study or country of publication.

### D. Selection of Study

The studies initially were screened using titles and abstracts. After finding a study with a relevant title and abstract, full screening was conducted. Furthermore, the relevant studies were considered after eliminating the possibility of type of publication, population and intervention. Moreover, bibliographies of the selected studies were checked for further studies not found in the initial search.

### E. Data Extraction and Synthesis

The data extraction was performed based on two main aspects: i) Datasets used in studies and ii) AI approach and architecture used. Table I demonstrates the detailed description of data description.

**TABLE I:** Data Extraction Format

**Datasets**	**Description**
Data Sources	Sources of data used in the studies for detecting COVID-19 via cough/breathing/speech recording – Public platforms, clinical settings, hospitals, government sources etc.
Data Size	The amount of data used to train, test and validate the algorithm
Data Collection	The method used for data collection such as open sources like apps or websites.
**AI Characteristics**	**Description**
AI Architecture	The architecture of AI algorithm such as CNN, RNN etc.
AI Branches	The type of AI model used such as: linear, ensemble, deep learning.
AI Platform	The platform used for AI processing such as computer or mobile.

After data extraction, the process of data synthesis for the selected studies was conducted. The data integration was concluded on the foundation of AI implementation. This included the type of AI architecture and its platform such as computer, tablets or mobile phones.

Moreover, the variety of branches used for the implementation of the models was also considered. On the other hand, the group of datasets were regarded as a tool for synthesis. These involved collection of data from various sources through multiple platforms. For instance, some studies obtained open-source recording of cough, breathing and speech through websites or mobile Apps. In addition, the sample size of the data used for training, testing and validating was also taken into account in this scoping review.

Although not used as a search criterion, another important consideration are cough, breathing and speech features extracted from the acoustic recordings of each dataset as well as their physiologic interpretation. These characteristics are summarized in Tables II and III for some of the included studies.

**TABLE II:** Features Used By Different Studies

**Papers**	**Features Used**
Thomas F. Quatieri	Formants(F1-F3), Fundamental Frequency (F0), Harmonic-to-noise ratio (HNR), Cepstral Peak Prominence, Creak, Envelope
Piyush Bagad	Log-Melspectrogram
Ali Imran	Mel Frequency Cepstral Coefficients (MFCC's), Mel-spectrogram
Lara Orlandic	MFFC's, Zero-crossing Rate, Energy Level, Spectral based features, Band Power, Signal strength, Power Spectral Density, Energy Envelope Peak
Jordi Laguarta	MFCC's
Ahmed Fakhry	MFCC's, Mel-spectrogram, Clinical information
Neeraj Sharma	MFCC's, Mean Square Energy, Polynomial Fit to the spectrum, Zero Crossing Rate, Spectral based features

**TABLE III:** A Physiological Interpretation of Features

**Feature**	**Physiology**	**Perception**
Formants	Vocal tract articulator movements	Defines consonant and vowel perception
MFCCs	Vocal tract articulator movements	Derived from spectrum of speech
Fundamental Frequency (F0)	Vocal fold vibrations	“Pitch” of speech
Harmonic-to-Noise Ratio (HNR)	Noise at the glottis	Hoarseness
Cepstral Peak Prominence (CPP)	Stability of vocal fold vibration	Jittery pitch
Creak	Compression of vocal folds – become slack and compact	Voice sounds like a creaky door, also seen as vocal fry
Speech Envelope	Contributions of the respiratory system and resonance-harmonics interaction to amplitude modulation of speech	Can be reflected in intensity or loudness of speech

## III. Results

### A. Search Results for Studies

Initially 86 studies were extracted and identified through a search in multiple databases and search engines. Out of these, 7 duplicates were eliminated for further screening. The titles and abstracts of the remaining 79 studies were screened. As a result, 41 studies were removed for reasons detailed in the Supplementary Material. The remaining 38 studies were examined by a full text review. However, 16 among these were excluded as they did not follow the study eligibility criteria. Consequently, 22 studies were incorporated. In addition, 2 more studies were identified and included after examining the reference list and reading literature reviews of the selected studies. Altogether, 24 studies and 8 websites/Apps were included in this review. websites/Apps which focused on cough, breathing or speech recordings were included (see Supplementary Material)

### B. Attributes of the Included Studies

Among the included studies, 8 were preprints and 16 were published articles in peer-reviewed journals (Table IV). Most of the studies were published between April 2020 - October 2020 and January 2021- July 2021. The included studies and Apps were conducted and launched in 12 countries. Nonetheless, more than half of these studies and applications, were from The USA, followed by India and U.K. with 6 studies and 2 applications respectively.

**TABLE IV:** Attributes of Studies and Applications Combined

ATTRIBUTES-Studies (n*=29)				
Paper Status-Studies (n*)	**Submission Months (for year 2020 and 2021 combined)-Studies (n)**		Country of Origin-Studies (n*)	
Published – 16	January – 3	July - 3	USA – 10	Luxembourg – 1
Preprint – 8	February – 1	August – 3	India – 7	Singapore – 1
Apps – 8	Mach – 2	September – 2	Switzerland – 1	Peru – 1
	April – 3	October – 2	Canada – 2	Russia – 1
	May – 2	November – 3	Germany – 2	UAE – 2
	June – 3	December – 0	U.K. – 3	South Africa – 1

Abbreviations: n = number of studies.

n^*^ = number of studies and applications.

### C. Characteristics of Datasets Utilized for AI Modelling

As shown in Table V, open-source public databases were the most commonly used resources for developing and modeling of AI architecture [11], [13]–[17], [21]–[27]. In addition, eight studies utilized both the clinical settings and publicly open datasets [13], [18], [20], [23], [27]. However, four of the included studies extracted the data from news interviews and social media platform such as Twitter, Instagram, Telegram or YouTube to train the AI algorithm [11], [20], [44].

**TABLE V:** Datasets of Included Studies (n) Only

**Data Sets-Studies (n=21)**		
**Data Set Size- Studies (n)**	**Data Types ^a^- Studies (n)**	**Data Sources ^b^- Studies (n)**
<1000 - 5	Cough recording - 15	Public databases - 14
1000 to 9999 - 11	Cough/Speech/Breathing recording – 6	Clinical settings - 8
≥ 10000 - 7	Speech recording – 6	Social Media - 4
N/A - 1	Cough/Breathing recording - 3	Interviews/News - 2
	Questionnaires - 3	Isolation Wards - 1
	Sound samples – 2	

a = Numbers do not add up as several studies collected more than one type of data.

b = Numbers do not add up as several studies collected their data from more than one data source.

**TABLE VI:** AI Architecture of Examined Studies (n)

**AIa Architecture (n=21)**			
**AI Branches b- Studies (n)**	**Input features**	**AI Algorithm c- Studies (n)**	**Platforms- Studies (n)**
Deep Learning –18	Cepstral Peak Prominence – 1	Convolutional Neural Network – 18	Computer/Websites –19
Other Machine Learning – 13	Mel- Spectrogram – 6	ResNet–50 Convolutional Neural Network – 4	Mobile – 4
	Mel – Frequency Cepstral Coefficients – 10	Rectifier Neural Network –2	

a = Artificial Intelligence.

b = Numbers do not add up because several studies have similar and more than one AI branch.

c = Numbers do not add up because several studies have similar and more than one AI architecture.

**TABLE VII:** COVID-19 Apps (N) Based on Cough, Breathing and Speech and Artificial Intelligence

**Apps/Websites (N=9)**		
**Stage Reached- Studies (N)**	**Data Stored ^a^ Types- Studies (N)**	**Platforms- Studies (N)**
Audio Collection – 8	Cough – 6	Website – 7
Diagnosis – 1	Speech – 6	Mobile – 2
Medical Advice – 2	Breathing – 2	
	Medical Questionnaire – 3	
	COVID-19 Symptoms – 3	

a = Numbers do not add up because several Apps have similar and more than one data stored types.

The type of data collected and stored as datasets were as follows: cough recordings (eg, recording for 3 s) [14], [15], [17], [23], [25]–[27] cough, breathing and speech recording (reciting a sentence) [16], [19], [22], [27]–[30], speech collection [11], [20], [21], [24] and sound samples (eg, recording using certain sounds) [18]. Moreover, some studies also collected a questionnaire based on a medical history or COVID-19 symptoms (eg, underlying conditions, age, sex, temperature) [15], [17].

The range of data size was observed from 5 to ≈32000. 10 of the studies used features like MFCCs [11], [13]–[18], [25]–[30] while 19 studies included CNN [11], [13]–[16], [18], [21]–[30] to extract features from the datasets. The data size was between 1000 and 9999 in half of the studies (n=11) [14], [16], [19], [22], [25]–[27], [28]–[30] whereas seven of the studies had the sample size greater than 10000 [15], [18], [20], [21], [28]. The Supplementary Material includes the datasets of all the examined studies.

### D. Characteristics of AI Architecture

Deep learning architectures were implemented in 18 studies [11], [13]–[20], [23], [27], [30]. On the other hand, one-third of the studies used both deep learning and non-deep learning architectures [15], [16], [19], [24], [28]–[30]. Four studies used branching architecture [17], [25], [29].

The most common AI model architecture recorded in more than half of the studies (n=18), was the Convolutional Neural Network (CNN) [11], [14], [16], [18], [20]–[30]. Six studies used signal and embedded processing techniques along with the Mel- Spectrogram [15], [19], [20], [29]. Four studies developed their algorithm by using ResNet-50 CNN which has a multi branching system providing a more robust architecture [17], [23], [25], [29].

Four studies implemented AI on a mobile platform [11], [19], [26], [30] whereas computer/desktop/websites were the platform in the remaining studies. However, in one of the preprints, no platform was specified [19]. The Supplementary Material section contains all the attributes and characteristics of AI architecture of the studies included.

### E. Search Results of COVID-19 Apps Based on Cough, Breathing and Speech Recordings

In the case of Apps and sponsored projects, all of them were at the audio collection stage [36]–[43]. On-board real-time AI processing had not yet been implemented in most systems.

However, one of the Apps developed gave a preliminary diagnosis of Influenza-like illnesses or COVID-19 screening utilizing features derived from cough, breathing and speech recordings [39] while two of the Apps recorded and collected cough data and returned recommendations for follow-up actions [40], [41].

Almost all the Apps (*n*=6) stored data in the form of cough, breathing and speech recordings. Nevertheless, 3 Apps required participants to fill in a medical questionnaire on the basis of recent medical history or any previous underlying condition to complete the data collection process [36], [37], [42].

In three included Apps, questions regarding COVID-19 symptoms were asked before the cough recording [39].

All the Apps (*n*=8) have their platforms on open-source websites. However, in two Apps the recordings can also be completed by using a mobile phone [36], [43]. The Supplementary Material provides the information of all the considered Apps.

### F. COVID-19 Positive With Symptoms/Asymptomatic and COVID-19 Negative (Dataset Characteristics of Studies)

**COVID-19 positive with symptomatic state:** In all the included studies, positive COVID-19 with underlying symptoms had an area-under-the-curve (AUC) performance ranging from 0.67 to 0.98. One-third of the studies recorded area-under-the-curve (AUC) performance of greater than 0.92 [11], [16], [17]. The highest accuracy to detect COVID-19 positive with cough was 98% shown in the two included studies [16], [20]. The accuracy percentages were recorded much lower in the studies where open-source databases/websites were used.

**COVID-19 positive asymptomatic state:** One of the studies achieved 100% sensitivity in predicting the diagnosis of individuals asymptomatic COVID-19 with features derived from recording collected in an open-source database [16].

**COVID-19 negative state:** Negative COVID-19 with cough and other symptoms were detected with an accuracy of greater than 70% in nearly all the included studies. In addition, area-under-the-curve (AUC) performances were greater than 0.80 in detecting negative COVID-19.

The accuracy of one of the Apps [36] was lower with a range from 77% to 80% with AUC of 0.79. Another App reported to return preliminary result within two minutes with an accuracy of 92.64% [11].

## IV. Discussion

In this study, a scoping review of AI detection of COVID-19 using cough, breathing and speech samples was conducted. The majority of the reported studies and launched Apps were published in the USA and India. Moreover, considering the lengthy publication process, one third of the included studies were preprints.

In the studies examined, the vital reason for AI modelling using cough, breathing and speech analysis was to identify the novel virus in asymptomatic patients and to make preliminary screening scalable, faster and reliable. The CNN architecture was used in most of the included studies. The most common platform used was computer based except for four studies [14], [19], [26], [30] that utilized mobile phones. Mobile phones can be used in more environments but with more background noise and this should be considered when observing a given model's performance.

Data sources in most of the included studies were crowd sourced (i.e., public datasets). Nonetheless, one study used news interviews and social media platforms for collecting voice samples which is likely to give low accuracy due to the disturbance and noise present in prerecorded samples. On the other hand, there were a few studies which were based on public databases or websites which collected more than 30000 recordings. However, there can be certain drawbacks in open-source databases such as discrepancy of recorded data, problems in recording device, incomplete recordings from the participants and lack of proper information in medical questionnaires. The cases of COVID-19 as of in August (2021) have surpassed 207 million [44].

### A. Practical and Research Implications

The COVID-19 cough detection concept was derived from previously implemented architectures such as in detection of Alzheimer's. Whilst this review examines the most common and recent architectures and features which have aided in detecting COVID-19 from cough, breathing and speech samples, there may be alternate architectures and features that could prove more useful in tackling problems and diseases that may arise in the future.

Although official tests such as RT-PCR or Serology for the novel virus have been widely employed, the results take hours, use up vital chemicals, can violate social distancing laws, take up time for a medical worker, are costly and can produce high false negatives. AI with cough/breathing/speech analysis may be a potential solution for preliminary screening eliminating the need of going to test centers, thereby saving time, maintaining social distancing rules with better efficiency and high reliability. Lastly, with the advancement of AI in mobile phones, this tool can be used as a daily screening measure in schools/colleges, workplaces and for everyday commuters to more rapidly prevent outbreaks or super-spreaders events. The type of the approaches covered in this review provides a non-invasive, free and real-time analyzing tool which could be beneficial in future for COVID-19 as well as other respiratory based diseases.

This review mainly focuses on publications and Apps that integrate AI architecture with cough, breathing and speech signals to detect COVID-19. However, further reviews may be required to evaluate the quality, validation and functionality of these AI algorithms.

### B. Strengths and Limitations

#### 1) Strengths

This review includes a published papers up to June 2021 and active Apps for AI in detecting COVID-19 through cough, breathing and speech sample regardless of their characteristics, study design, study setting, and country of publication. This study shows the significance of AI in analyzing and detecting a crucial heath related issue. This review follows the full scientific rigor of PRISMA-ScR. Additionally, the risks of duplicate publication were minimized by using Google Scholar along with other search engines. Furthermore, we used the reference sections of all identified journal papers to ensure that our search strategy was not missing any relevant publications.

#### 2) Limitations

The search study was restricted to English due to practical constraints. Due to this reason, some publications written in other languages might be missed, especially Chinese. The search terms were generalized such as artificial intelligence, COVID-19 cough, breathing and speech etc. but not the AI model names such as CNN, RNN etc. Lastly, some papers are preprints which may affect the accuracy of the information in the included studies.

## V. Conclusion

In this manuscript, we provide a scoping review of 24 studies and 8 Apps for detecting COVID-19 by cough, breathing and speech recording using AI algorithms. Given, the initial results from multiple studies, this will be a promising research area, since a successful application would save time, reduce scarcity for official testing in small countries and maintain social distancing. In addition, with broad training, testing and validation of artificial intelligence, along with the neurophysiological understanding of human body; the proposed methods could bring a big difference in the fight against COVID-19 and future pandemics.

## Supplementary Materials

Supplementary materials
