# Supplementary material for: Artificial Intelligence for Detecting COVID-19 With the Aid of Human Cough, Breathing and Speech Signals: Scoping Review
Source: IEEE Open J Eng Med Biol. 2022 Feb 14;3:235–41. doi: 10.1109/OJEMB.2022.3143688 (PMC9933914; doi:10.1109/OJEMB.2022.3143688)

Supplementary Materials

Artificial Intelligence for Detecting COVID-19 with the Aid of Human Cough, Breathing and Speech Signals: Scoping Review

Mouzzam Husain, Andrew Simpkin, Claire Gibbons, Tanya Talkar, Daniel Low, Paolo Bonato, Satrajit Ghosh, Thomas Quatieri, Derek T.O' Keeffe

Table 1: Datasets for development of AI

Author	Data source	Data type	Dataset size
**Quatieri**	Social Media, Interviews	Voice/Speech recording	5
**Bagad**	Clinical Settings, Isolation wards	Cough-Voice recording	3,621
**Imran**	Public database	Cough recording	5,435
**Orlandic**	Public database	Cough recording, questionnaire	>20,000
**Laguarta**	Public database	Cough and voice recordings	4,256
**Fakhry**	Clinical Setting	Cough recording, questionnaire	5,749
**Sharma**	Clinical setting	Sound samples	>20,000
**Hassan**	Clinical setting	Cough, breathing and speech recordings	240
**Faezipour**	Public database	Oxygen, Lung capacity	N/A
**Ritwik**	Social media, Interviews	Speech recordings	19
**Dash**	Public database, Clinical setting	Speech recordings	>20,000
**Mouawad**	Public database	Cough, breathing and speech recordings	3,415
**Andreu-Perez**	Clinical setting	Cough recordings	8,380
**Wei**	Clinical setting	Speech recordings	1,283
**Pahar**	Public database	Cough, breathing and speech recordings	5,816
**Coppock**	Public database	Breath and cough recordings	517
**Rodriguez**	Clinical setting	Cough, breathing and speech recordings	3,716
**Ponomarchuk**	Public database	Cough, breathing and speech recordings	3,409
**Mohammed**	Public database	Cough recordings	>10,000
**Kumar**	Public database	Cough, breathing and speech recordings	7,000
**Pal**	Clinical setting, Public database	Cough and voice recordings	>30,000
**Alqudaihi**	Clinical setting	Cough, breathing and speech recordings	<10,000
**Brown**	Public database	Cough, breathing and speech recordings	>10,000

Table 2: Attributes and AI architecture

Author	Submission month	Country	Paper status	AI branch	AI models/ algorithms	Platform
**Quatieri**	April	USA	Published	DL	CPP,HNR,F1-F3,F0	COMPUTER
**Bagad**	September	India	preprint	DL	CNN, ReLU	COMPUTER
**Imran**	June	USA	Published	DL	CNN,MFCC	MOBILE
**Orlandic**	September	Switzerland	Preprint	DL, ML	SP,MFCC	COMPUTER
**Laguarta**	August	USA	Published	DL, ML	CNN,MFCC	COMPUTER
**Fakhry**	March	USA	Preprint	ML	ResNet-50 CNN,MFCC	COMPUTER
**Sharma**	August	India	Preprint	DL	CNN, MFCC	COMPUTER
**Hassan**	November	UAE	Published	DL	MFCC, RNN	COMPUTER
**Faezipour**	October	USA	Preprint	DL, ML	Unspecified	MOBILE
**Ritwik**	November	India	Preprint	DL	Mel-Spectrogram, MFCC	COMPUTER
**Dash**	April	India	Published	DL	MFCC	COMPUTER
**Mouawad**	January	Singapore	Published	DL	MFCC	COMPUTER
**Andreu-Perez**	February	UK	Published	DL	CNN	COMPUTER
**Wei**	August	China	Preprint	ML	CNN	COMPUTER
**Pahar**	June	South Africa	Preprint	ML	CNN, ResNet-50	COMPUTER
**Coppock**	April	UK	Published	ML	CNN	MOBILE
**Rodriguez**	November	Peru	Published	DL, ML	CNN	COMPUTER
**Ponomarchuk**	July	Russia	Preprint	ML	Mel-Spectrogram, CNN	COMPUTER
**Mohammed**	July	Canada	Published	ML	CNN, ResNet-50	COMPUTER
**Kumar**	May	India	Published	DL, ML	CNN	MOBILE
**Pal**	October	India	Preprint	DL	SP,EP,PY	COMPUTER
**Alqudaihi**	January	India	Published	DL, ML	CNN	COMPUTER
**Brown**	July	USA	Published	DL, ML	CNN	COMPUTER

Table 3: Apps / Projects

App Name	Stage	Nature of Data Stored	Country/University	Platform
**COVID-19 Sounds**	Audio Collection	Cough, breathing, voice, questionnaire	University of Cambridge, UK	Website/Mobile
**Breathe for Science**	Audio Collection	Cough, questionnaire	New York University, USA	Website
**COVID voice Detect**	Audio Collection	Voice recording	Carnegie Mellon University, USA	Website
**VoiceMed**	Audio Collection, Diagnosis	Cough, breathing, voice	Luxembourg	Website
**DetectNow**	Audio collection, medical advice	Cough, COVID-19 symptoms	USA	Website
**COVID-19 Voice study**	Audio collection, medical advice	Voice	USA/Canada	Website
**Novoic**	Audio Collection	Cough, voice, questionnaire	UK	Website
**Hyfe Cough**	Audio Collection	Cough	USA	Mobile

Study Selection Process Flowchart

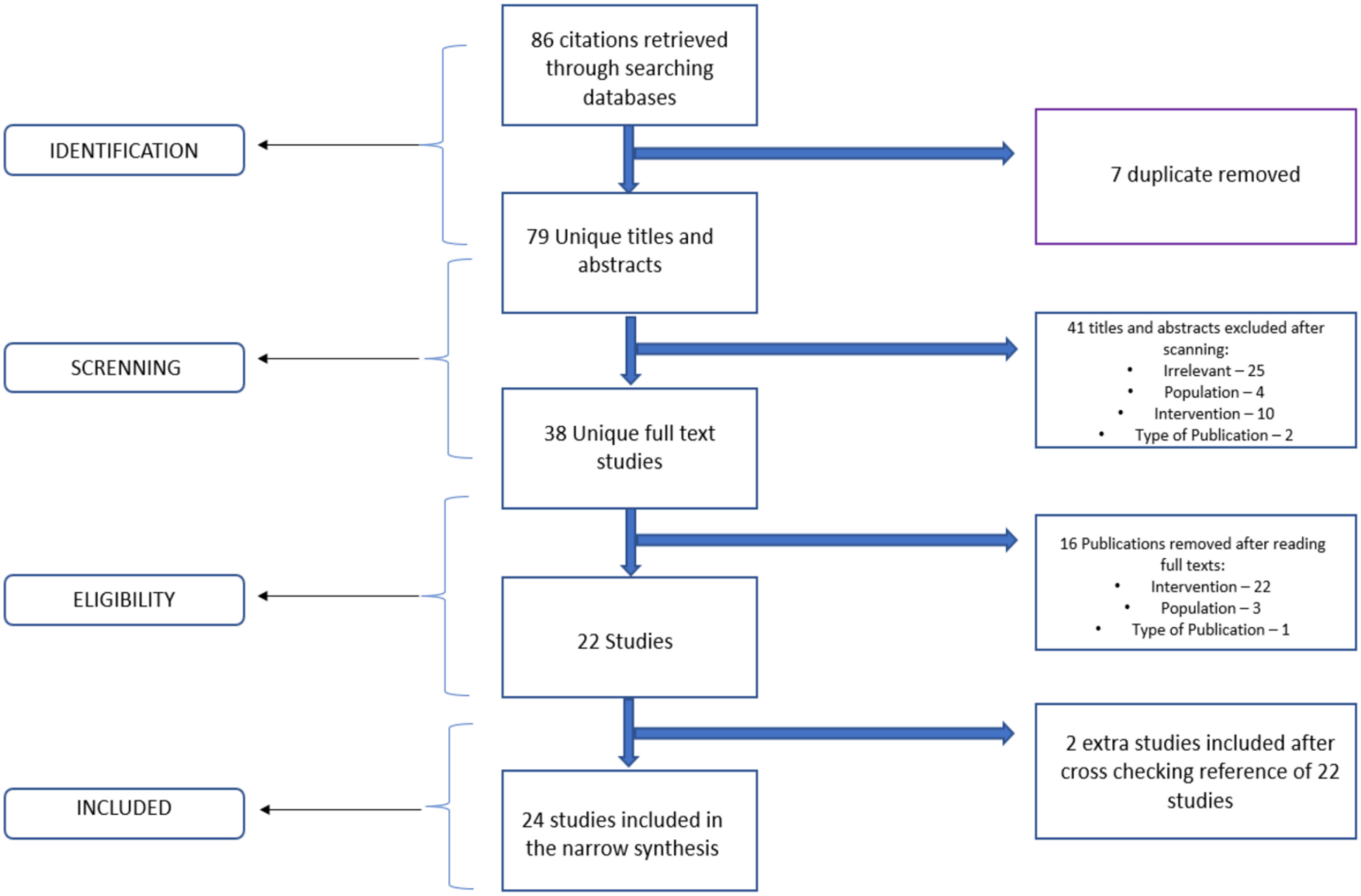

Supplement: Supplementary materials [file supp1-3143688.pdf]
